# Cross-interference of hemolysis, icterus and lipemia: A study with real patient samples

**DOI:** 10.11613/BM.2026.020708

**Published:** 2026-06-15

**Authors:** Hatice Bozkurt Yavuz, Soycan Tüner

**Affiliations:** Department of Clinical Chemistry, Uşak University Faculty of Medicine, Uşak, Turkey

**Keywords:** hemolysis, icterus, lipemia, preanalytical phase, interference, sample handling

## Abstract

**Introduction:**

Hemolysis, icterus, and lipemia (HIL) are frequent preanalytical interferences, leading to erroneous results or sample rejection. While individual effects of these indices have been studied, their interferences on each other remain largely unexplored. This study aimed to evaluate the cross-interference of HIL parameters using endogenous patient-derived samples, thereby ensuring clinically relevant conditions, and to propose a decision algorithm for improved sample quality assessment.

**Materials and methods:**

Following the CLSI-EP07 guideline, high-concentration icterus and lipemia pools from leftover clinical samples and hemolizate pool have been prepared. These pools were serially diluted with low-index serum (HIL0) to obtain defined combinations. Indices were measured using the Abbott Alinity c system. Cross interferences were calculated as percentage bias compared to baseline values.

**Results:**

High level hemolysis remained largely unaffected by other indices. Hemolysis in the range of 2.5-10.0 g/L caused a 20-150% false increase in lipemia and a 10-60% false decrease in icterus. Conversely, lipemia in the range of 25-100 index led to a 20-150% false increase in icterus and reduced low level of hemolysis by 10-30%. Similarly, icterus at 42.75-171 µmol/L resulted in a 15-45% false reduction in low levels of hemolysis. No concentration-dependent systematic bias was observed for lipemia and icterus, whereas hemolysis showed level-dependent effects.

**Conclusions:**

Cross-interference among HIL indices can lead to misinterpretation if indices are assessed independently. A rule-based, threshold-dependent algorithm incorporating these interactions is proposed to enhance accuracy in sample rejection decisions. Implementation of such integrated approaches, along with manufacturer transparency may significantly improve laboratory reliability.

## Graphical abstract

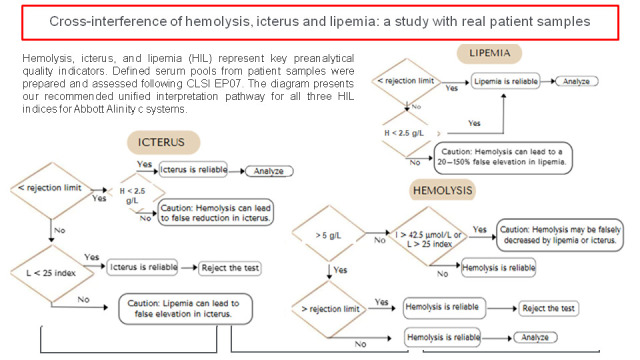

## Highlights

• Hemolysis, icterus and lipemia (HIL) are among the most common errors encountered in the preanalytical phase

• Cross-interference among HIL indices can cause misinterpretation

• Hemolysis falsely increased lipemia and decreased icterus measurements

• Lipemia led to false increases in icterus and reduced mild hemolysis

• Icterus caused a false reduction in low levels of hemolysis

## Introduction

Hemolysis, icterus, and lipemia (HIL) are frequent preanalytical issues that can interfere with routine test results and lead to misinterpretation (*1*). Hemolysis, icterus, and lipemia were previously assessed visually based on color and turbidity, but this method was time-consuming, subjective, and lacked standardization (*2*, *3*). The Clinical and Laboratory Standards Institute (CLSI) C56A guideline recommends the use of a detection system for HIL serum indices to ensure reliable assessment of sample quality (*4*).

Hemolysis, icterus, and lipemia are among the primary rejection criteria used in clinical laboratories. Moreover, *in vitro* hemolysis is the most common error, affecting up to 3.3% of routine blood samples and representing 40-70% of all unsuitable specimens (*1*). The cut-off values provided by reagent manufacturers are commonly used in test rejection protocols; however, reagent package inserts generally do not include information on the mutual interference between HIL parameters (*5*, *6*). This may lead to the performance of a test that should have been rejected or the rejection of a test that should have been analyzed. There are studies showing that hemolysis affects icterus and that lipemia increases hemolysis (*7*, *8*). However, to the best of our knowledge, no study has evaluated the mutual interference of all three parameters - hemolysis, icterus, and lipemia. Recent research emphasizes the importance of using natural lipemic samples in interference studies to improve the accuracy of results and facilitate effective patient management (*9*). The purpose of this study is to assess the cross-interference of HIL parameters under clinically relevant conditions by using only endogenous patient samples and to develop a decision algorithm for improving sample quality evaluation.

## Materials and methods

This experimental analytical interference study was conducted in accordance with the CLSI EP07 guideline. This guideline recommends evaluating the effect of hemolysis, icterus, and lipemia at five different concentrations (including zero) on two different concentrations of the target analyte (*10*). While this approach is relatively straightforward for a standard analyte routinely measured in laboratories, our study aimed to investigate the mutual effects of hemolysis, icterus, and lipemia on each other. Therefore, a modified approach was adopted to align with this objective. The reason for this adjustment is that the added interfering substances themselves affect the measured outcome. To address this, serial dilutions of the interfering substances were performed, their recoveries calculated, and comparisons were made using these target values. The analyses were performed using the Abbott Alinity c system (Abbott Laboratories, Chicago, USA). The study was conducted with the approval of the Uşak University non-interventional studies ethics committee (778-778-20).

### Sample selection and sample preparation

A serum pool with hemolysis < 0.02 g/L, lipemia < 2 index and icterus < 20 µmol/L was prepared from leftover samples to be used as a diluent and was designated as HIL0. The HIL0 pool was prepared using samples from 10 patients. To create lipemic and icteric serum pools, sera from patients with high lipemia and icterus levels were used. For the icterus pool, sufficient serum was obtained from two patients with cholestasis, whereas the lipemia pool was prepared using sera from three different patients. The hemolysate-containing pool was prepared by generating hemolysate from the complete blood count samples of a patient whose serum sample was used in the HIL0 pool, thereby ensuring the absence of lipemia and icterus, with freezing/thawing of whole blood followed by the osmotic shock protocol (*11*). Because the hemolysis level was very high (> 100 g/L), the hemolysate was diluted with HIL0 serum to achieve the desired (2 g/L) hemoglobin concentration.

Samples were considered eligible for the high-lipemia pool if they showed a lipemia index of 300 with hemolysis < 0.02 g/L and icterus < 20 µmol/L. Similarly, samples were included in the high-icterus pool if they had an icterus level of 228 µmol/L with hemolysis < 0.02 g/L and lipemia < 2 index. Samples showing concurrent elevation of more than one HIL parameter were excluded from pool preparation.

The HIL-0 and high-concentration pools were prepared from fresh samples that had completed analysis on the day of the study, and no freezing, thawing, or prolonged storage was performed. Serial dilutions of these high-concentration HIL pools with the HIL0 pool showed no significant deviation, which simplified the study; the results obtained from the serial dilutions, with each sample analyzed in five replicates, are presented in Table 1. As the obtained values were closely clustered, no outliers were identified. This allowed for the calculation of expected HIL values in serum pools mixed in known ratios, enabling comparison with measured HIL values. For example, to obtain a pool containing 5 H and 171 I, 1 mL of the H 20 pool and 3 mL of the I 228 pool were mixed, resulting in a 4 mL sample with 5 H, 171 I, and L < 2. The H value was measured in this sample, allowing the interference of 171 icterus on 5 H to be determined.

**Table 1:** Serial dilution results of high index pools with HIL0 serum sample

**High-index: HIL0 ratio**	**Number of replicates**	**Expected H index** **(g/L)**	**Measured H index (g/L)**	**Expected I index** **(µmol/L)**	**Measured I index (µmol/L)**	**Expected L index** **(index)**	**Measured L index (index)**
1:0	5	20	20 ± 0.6	228	228 ± 6.9	300	300 ± 10
1:1	5	10	10 ± 0.38	114	114 ± 6.7	150	151 ± 6
1:3	5	5	5.02 ± 0.18	67	66.7 ± 2.6	75	74 ± 3
1:5	5	3.33	3.29 ± 0.10	38	38.1 ± 2.4	50	51 ± 2.5
1:9	5	2	2.01 ± 0.06	23	22.8 ± 1.7	30	29 ± 1.5
H - hemolysis. I - icterus. L - lipemia. HIL0 - hemolysis, icterus, lipemia free serum sample. The measured H, I and L index are presented as mean and standard deviation.							

The prepared interference tubes were labeled and briefly vortex-mixed, then arranged in a random order on the analyzer racks. Prior to each analytical run, vortexing was repeated, and the tubes were re-randomized to ensure that the order of analysis differed from the previous run.

### Acceptable interference limit

The guideline recommends the use of total allowable error when defining acceptable interference limits; however, such limits are not defined for hemolysis, icterus, and lipemia indices in the European Federation of Clinical Chemistry and Laboratory Medicine (EFLM) Biological Variation database. In practice, reagent package inserts commonly apply an arbitrary threshold of 10% as the allowable amount of interference (*6*). In the present study, the outcome variables were the serum indices themselves (hemolysis, icterus, and lipemia) rather than patient-reportable analytes. Therefore, analyte-specific clinical interference limits based on total allowable error were not directly applicable in this context, and the analysis focused on the analytical interaction between the indices. In our study, considering that hemolysis can affect clinically critical analytes such as potassium even at low levels, we predefined an allowable difference of 10%. Accordingly, results showing changes exceeding ± 10% and demonstrating a consistent directional pattern in accordance with the monotonicity concept described in the guideline were interpreted as false-positive or false-negative results.

### Number of replicates

According to CLSI EP07, when the ratio of the allowable difference (δ) to within-run imprecision (σ) exceeds 2, a minimum of five replicates is recommended. In this study, the allowable interference was predefined as 10%, the coefficient of variation (CV) for hemolysis, icterus, and lipemia were 3.0%, 3.7%, and 4.2%, yielding δ/σ ratios of 3.33, 2.7, and 2.4, respectively. As all ratios exceeded 2, five replicate measurements were performed for each dilution level and index.

### Statistical analysis

Cross interferences were calculated as percentage bias compared to baseline values using the formula: Bias (%) = [(Measured Value - Expected Value) / Expected Value] x 100. Within-run imprecision was evaluated using CV (%). Data points from the five replicates were expressed as mean percentage differences, and descriptive statistics (mean ± standard deviation) were calculated using Microsoft Excel .

## Results

**Figure 1:**
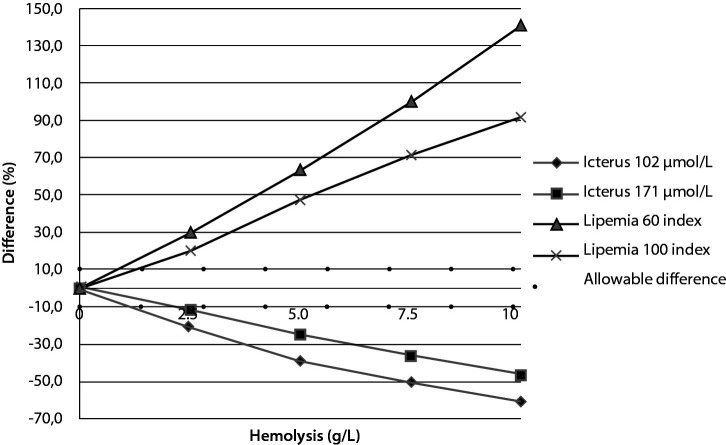
Percent difference of icterus and lipemia indices under hemolysis interference. Each sample was analyzed in five replicates; data points represent mean percentage differences.

**Figure 2:**
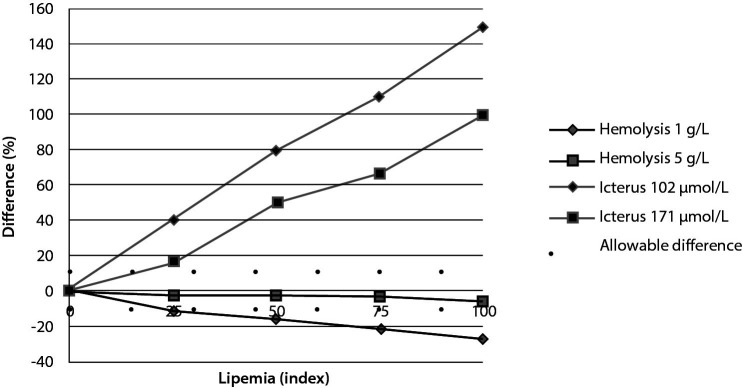
Percent difference of icterus and hemolysis indices under lipemia interference. Each sample was analyzed in five replicates; data points represent mean percentage differences.

**Figure 3:**
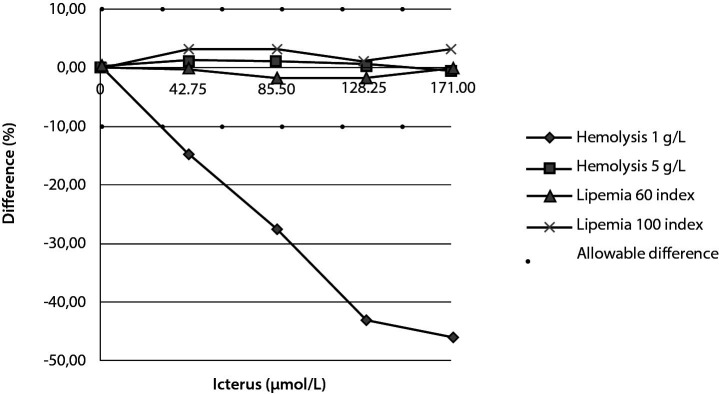
Percent difference of hemolysis and lipemia indices under icterus interference. Each sample was analyzed in five replicates; data points represent mean percentage differences.

An increase in hemolysis leads to an increase in lipemia results, while a decrease is observed in the icterus. Figure 1 summarizes the percentage increases and decreases relative to the baseline value. The low level of icterus is the index level most positively affected by lipemia, with an observed increase of up to 150%. In contrast to icterus, lipemia decreases hemolysis. The effect of increasing lipemia on hemolysis and icterus is summarized in Figure 2. Although icterus does not have a significant effect on the results in the presence of lipemia and high-level hemolysis (bias < 10%), it causes a nearly 50% decrease in low-level hemolysis when the icterus level reaches 171 µmol/L. The effect of icterus on hemolysis and lipemia is summarized in Figure 3. Lipemia was not influenced by icterus, and false increases in the lipemia index occurred only at hemolysis levels above 2.5 g/L, while no false decrease was observed, indicating that low lipemia index values remain reliable.

## Discussion

In this study, we proposed a decision algorithm based on HIL indices, taking into account the mutual interactions among them. High hemolysis values are considered reliable, as no interference from other indices leads to a false increase in the hemolysis index. Similarly, low lipemia index values are deemed accurate, as they are not subject to falsely decreased results due to other indices.

**Figure 4:**
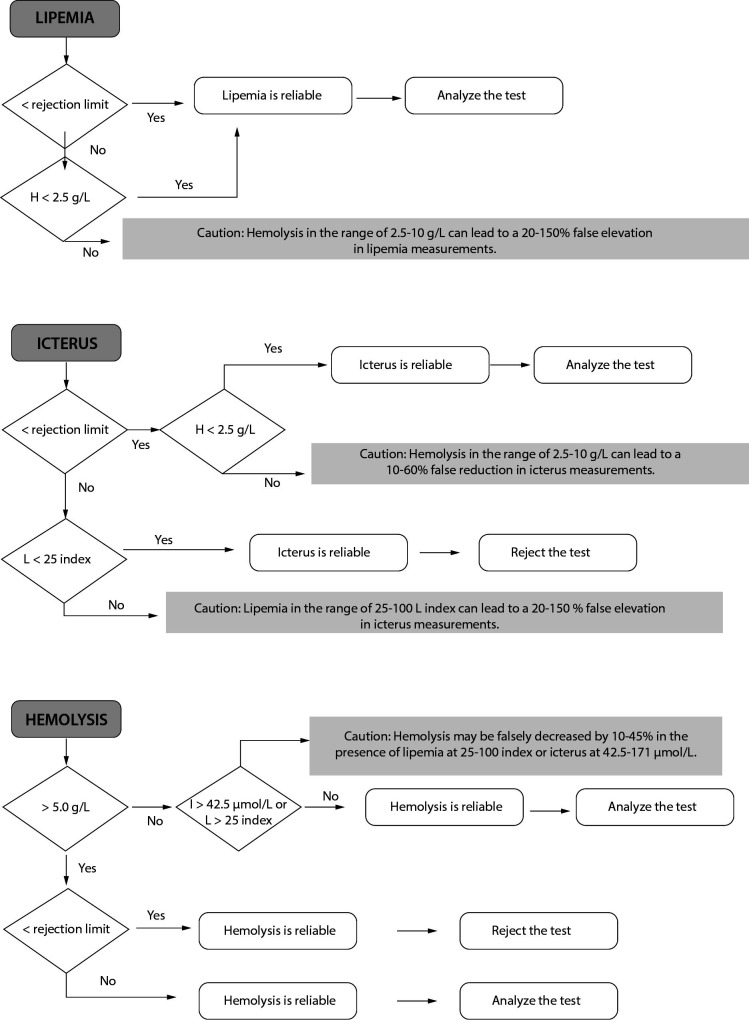
Hemolysis, icterus, lipemia - based decision algorithm incorporating cross-interference effects on Abbott Alinity c systems. Platform-specific hemolysis-, icterus and lipemia-based decision algorithm incorporating cross-interference effects for Abbott Alinity c systems.

For lipemia and icterus, we observed no concentration-dependent systematic shift in interference and therefore developed a rule-based algorithm grounded in individual test-specific rejection thresholds for Abbott Alinity c system. However, a different approach was necessary for hemolysis, since high and low levels of hemolysis were not equally affected by icterus and lipemia interference. Based on this observation, we stratified hemolysis using a threshold of 5 g/L, and created a conditional algorithm accordingly. This was particularly relevant for analytes sensitive to even low-level hemolysis. The final step of the proposed algorithm requires expert review, as introducing a fixed correction factor was not feasible due to the concentration-dependent nature of HIL effects. Therefore, instead of applying a universal mathematical correction, we prioritized a structured interpretive approach that integrates both laboratory thresholds and expert opinion. A summary of the proposed algorithm is presented in Figure 4.

In tests that are affected even by low levels of hemolysis, the simultaneous presence of hemolysis and icterus may lead to the unintentional reporting of a result that should have been rejected. For instance, in a patient presenting with hyperbilirubinemia (total bilirubin concentration: 171 µmol/L), if the hemolysis index is measured as 0.9 g/L by the analyzer, the result may be reported since it falls below the rejection threshold of 1.25 g/L hemoglobin, defined in the kit insert for the potassium assay (*12*). However, considering that the icterus index is 171 µmol/L in this case, our findings indicate that the actual hemolysis may exceed the rejection threshold, 1.25 g/L hemoglobin, and thus the result should not be reported. This may lead to an artificially elevated potassium concentration, potentially altering and prolonging the course of treatment.

The need for greater transparency from manufacturers regarding HIL interference has previously been emphasized by the EFLM Working group for preanalytical phase (*13*). However, upon reviewing the Abbott Alinity c package inserts, we found that certain reagent inserts do not provide detailed information on how lipid interference studies are conducted, and some do not even specify the hemolysis concentrations that affect the result (*14*, *15*). In our view, this transparency should include detailed diagrams explaining both the units used and the methodology of the study, and the evaluations should be applicable to real patient samples rather than being based solely on artificial substances such as Intralipid.

On the other hand, the use of Intralipid solution is common in lipemia studies, and certain Abbott kit package inserts also evaluate lipemic interference using Intralipid (*5*). In a study evaluating the effects of HIL on analytes, the measured concentrations of hemoglobin and total bilirubin were used to represent hemolysis and icterus, respectively, while the calculated concentration of Intralipid was used for lipemia, and serial dilutions of the Intralipid solution were reported to align with Abbott index values when expressed in mg/dL (*2*). The degree of turbidity does not always correlate well with triglyceride concentration; although a strong linear relationship has been shown using standardized lipid emulsions, this correlation is much weaker in real patient samples (*16*). Kit manufacturers may report interference evaluations for lipemia up to 10 g/L Intralipid concentration in their package inserts (*5*). However, in the lipemia part of our study, the highest achievable lipemia value was 300 index, and the leftover samples used to prepare this pool had triglyceride concentrations exceeding 20 g/L. These values were among the highest identified when we retrospectively reviewed the laboratory information system. The difference between Intralipid and native lipemia may be attributed to the fact that the refractive index of Intralipid particles differs from that of lipoproteins (*9*). Therefore, in our study, we chose to use g/L units for hemoglobin and µmol/L for icterus, while using unit of ‘index’ for lipemia, as it did not correlate with real triglyceride concentration.

This study has certain limitations. While preparing the HIL0 pool, it was not possible to identify a sample in which the HIL indices were all simultaneously zero. As a result, there may be minimal interference present in the baseline pool. However, considering the very high concentrations of the added interferents, we believe that such a small magnitude of variation in any index would have a negligible impact on the overall results. In addition, during the preparation of the hemolysate, the selected samples were processed with distilled water. Thus, the only artificial substance used throughout the study was distilled water. Regarding the lipemia effect on hemolysis, although the patient sample used in the hemolysate preparation was not lipemic, the L index was found to be elevated, in line with our study hypothesis. Since there was no lipemia in the sample, L < 2 was accepted in the hemolysate without additional lipemia and the increase due to lipemia addition was calculated accordingly. Also, all measurements were performed on a single analytical platform (Abbott Alinity c); therefore, the observed HIL index behavior and the proposed decision algorithm are platform-specific, and the findings may not be directly generalizable to other analytical systems.

In conclusion, this study highlights that HIL indices can influence not only test results but also each other’s measurements. These mutual interferences indicate that evaluating HIL indices in isolation may lead to misinterpretation, especially in borderline cases. Therefore, integrated algorithms that consider cross-interference effects and provide expert-based interpretation are essential for improving the accuracy and reliability of laboratory test processes.

## Data Availability

All data generated and analyzed in the presented study are included in this published article.
